# Supplementary material for: Nanopore electric snapshots of an RNA tertiary folding pathway
Source: Nat Commun. 2017 Nov 13;8:1458. doi: 10.1038/s41467-017-01588-z (PMC5684407; doi:10.1038/s41467-017-01588-z)
Supplement: Supplementary file 1 — Supplementary Information [file 41467_2017_1588_MOESM1_ESM.pdf]

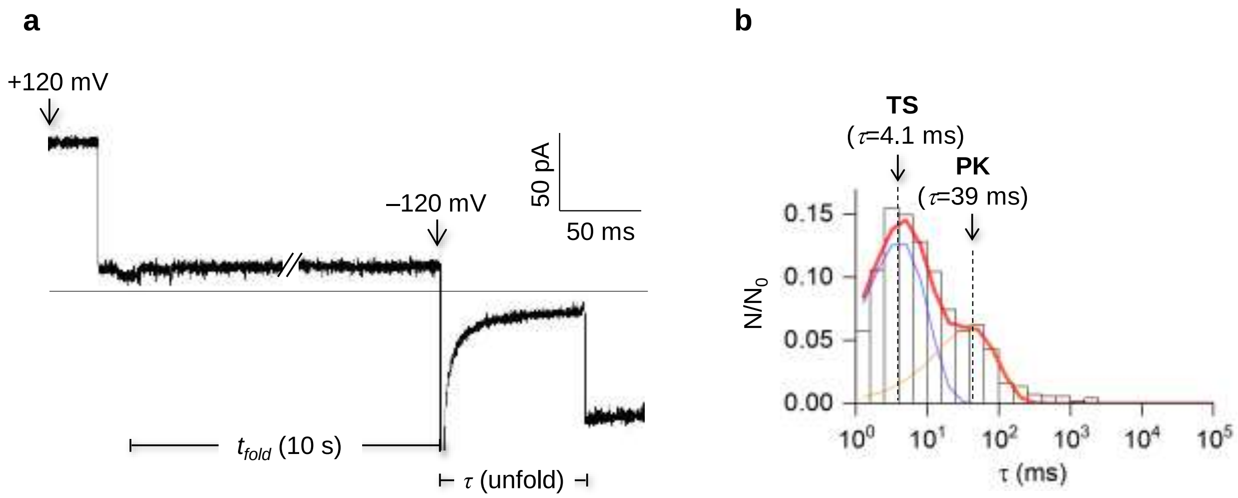

Supplementary Fig. 1. Detection of folding states at –120 mV. **a.** Current trace showing the trapping, folding and disruption of folding states of T2 RNA. The complex probe was trapped and immobilized in the nanopore at +120 mV. After folding for t_{fold}=10 s, the voltage was changed to –120 mV to disrupt the folding structure. The lifetime (τ) of that folding state was the duration between the beginning of voltage (–120 mV) application and the block end. **b.** Histogram showing the distribution of lifetime (N_0=756) and the fitting of the distribution (Eq. M2 in Methods) with two states, TS and PK. Both states were greatly shortened compared with that at –60 mV. The very short HP1 and HP2 states that were observed at –60 mV (Fig. 2 in main text) were not seen at –120 mV, suggesting that low voltage is a unique and powerful tool for identifying short intermediate structures.

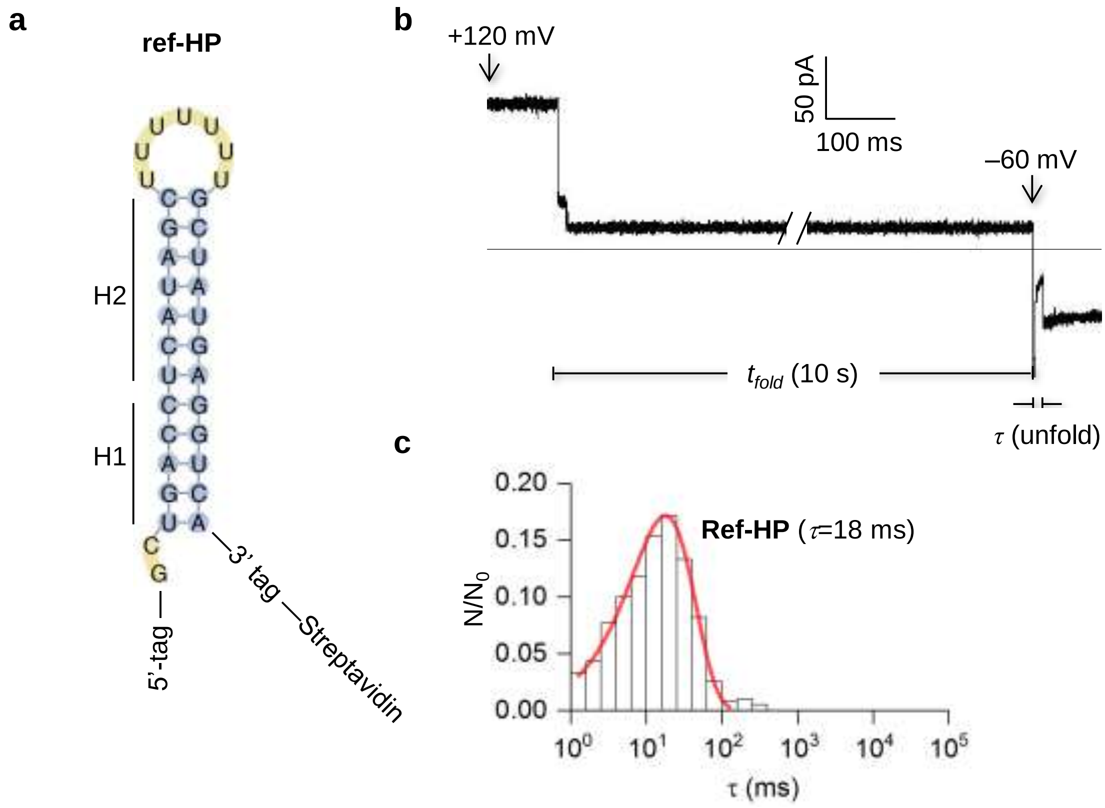

Supplementary Fig. 2. Reference RNA hairpin ref-HP. **a.** 2D structure of Ref-HP. The sequence of T2-RNA was re-arranged to construct this reference RNA (Table 1 for the sequence), such that it no longer formed a pseudoknot but could only form a 12-bp hairpin. This hairpin links H1 (5 bps) and H2 (7 bps) of the native pseudoknot together, but the original inter-helix loop L2 is removed. This reference hairpin is also linked with a poly$(CAT)_{10}$ DNA tag at both 5' and 3' ends, and the 3' tag is attached to a streptavidin. **b.** Nanopore current signature showing the folding and unfolding of the Ref-HP hairpin structure. Ref-HP was characterized using the same +120 mV/t_{fold}=10 s/−60 mV protocol as T2 RNA: the polymer was trapped and immobilized in the pore at +120 mV; then, the RNA refolded on the *trans* side to form a 12-bp hairpin. After a given folding time (t_{fold}=10 s), a voltage of –60 mV was applied to unfold the hairpin. c. Histogram showing the distribution of lifetime (N_0=383) and the fitting of the distribution. Our nanopore force spectroscopy results also show that unlike T2 pseudoknot unfolding in the *trans*

side, ref-HP's unfolding duration was a single component, with τ=18 ms ± 3 ms at –60 mV. This result indicates that as the loop-helix interaction is removed, the stability of ref-HP is much lower than the pseudoknot that also forms a 12-bp hairpin and should be contributed by the 12 base pairs only. Ref-HP was used as a marker to compare with the intermediate folding states. The differences in their unfolding durations can suggest possible structures such as base pair numbers and loop interactions in the intermediate states.

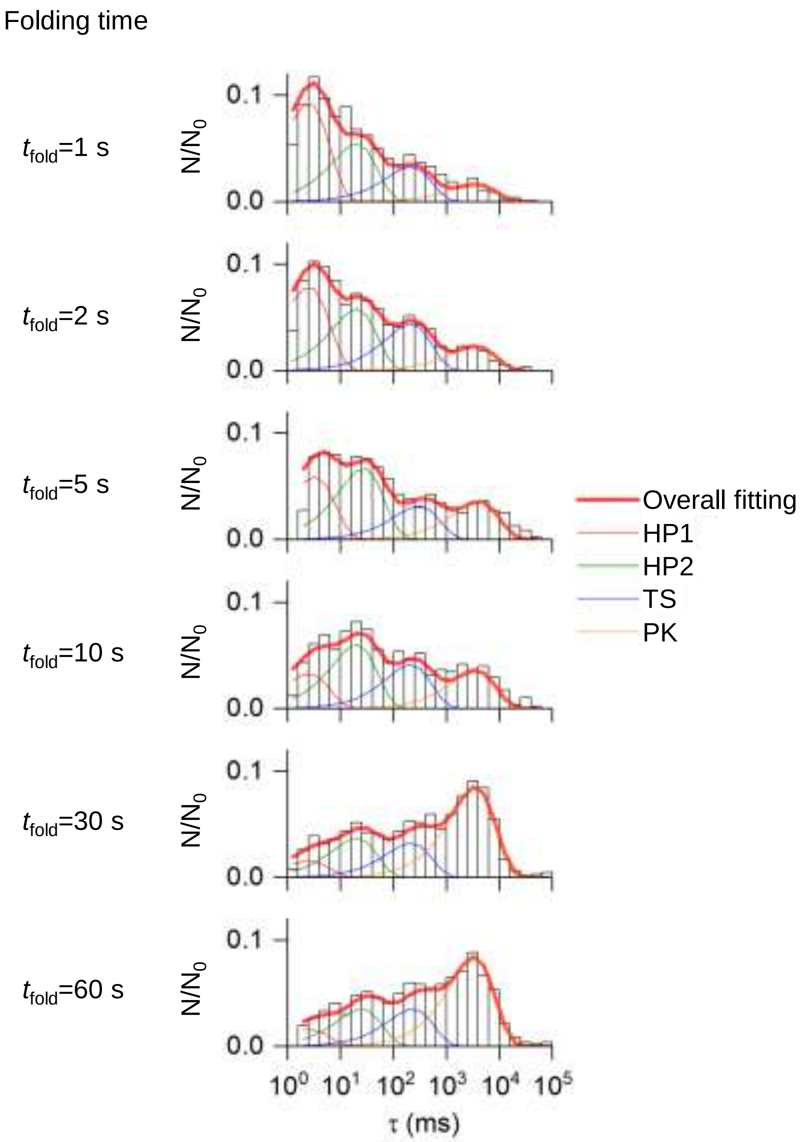

Supplementary Fig. 3. RNA unfolding duration distribution at various folding times. These distribution histograms were obtained after folding for t_{fold}=1 s (N_0=548), 2 s (N_0=568), 5 s (N_0=476), 10 s (N_0=578), 30 s (N_0=644) and 60 s (N_0=567). Each histogram was fitted with four components, HP1, HP2, TS and PK, using the protocol described in the Methods (Eq. M2). The fractional population of each folding state (P) was calculated based on the area under the corresponding peak in the histogram using the protocol described in the Methods (Eq. M3).

Calculated state probabilities were plotted versus the folding time in Fig. 4a in the main text. Folding time-dependent state populations enable the establishment of folding pathways and calculating the transition rates, as shown in Fig. 4b.

a

Chimera name	Structure and sequence
dC35	5′-CCCCCCCCCCCCCCACCCCCCCCCCCCCCCCCCCC-3′-Biotin
dC5-rC15	5′-CCCCCCCCCCCCCCACCCCCCCCCCCCCCCCCCCC-3′-Biotin
dC14-rC5	5′-CCCCCCCCCCCCCCACCCCCCCCCCCCCCCCCCCC-3′-Biotin
dC11-rC5	5′-CCCCCCCCCCCCCCACCCCCCCCCCCCCCCCCCCC-3′-Biotin
dC8-rC5	5′-CCCCCCCCCCCCCCACCCCCCCCCCCCCCCCCCCC-3′-Biotin
dC5-rC5	5′-CCCCCCCCCCCCCCACCCCCCCCCCCCCCCCCCCC-3′-Biotin

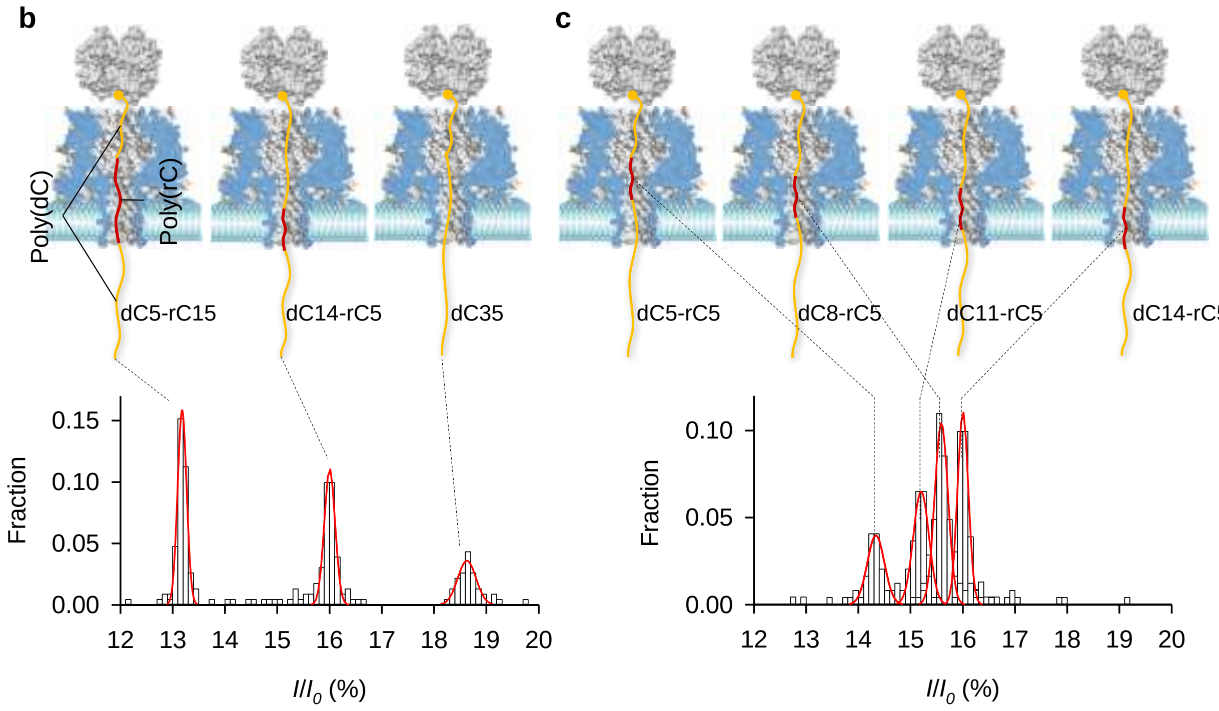

Supplementary Fig. 4. Nanopore discrimination of RNAs in DNA-RNA chimeras. **a.** Sequences of a group of poly-cytosine DNA-RNA chimeras. All chimeras are 35 nt long and 3'-biotinylated. Fragments in red are poly(rC) RNA, and other fragments in yellow are poly(dC) DNA. The RNA lengths and/or positions in these polymers are different from each other. The entire nanopore can accommodate approximately 20 nucleotides[1], and the underlined fragment between the 10th and 20th nucleotides from the 3' end occupies the narrow stem (β-barrel) of the αHL pore, which

dominates the nanopore ionic conductance. **b.** Cartoons showing the RNA positions (top) and blocking level histogram (bottom) showing the discrimination of streptavidin-attached chimeras dC35, dC5-rC15, and dC14-rC5 trapped in the nanopore. Blocking level was measured as I/I_0, where I and I_0 are the current amplitudes of the blockade and empty pore, respectively. As dC35 does not contain an RNA component, the pore stem is fully occupied by poly(dC) DNA, generating the highest blocking level (I/I_0=18.6%) among the three chimeras. In contrast, dC5-rC15 contains a 15-nt poly(rC) RNA that is long enough to thread the entire pore stem, giving rise to the lowest blocking level (I/I_0=13.2%). By comparison, dC14-rC5 possesses a 5-nt poly(rC) RNA. This short RNA fragment is expected to occupy half of the pore stem near the *trans* entrance, while the other half is occupied by the poly(dC) DNA. The blocking level for such a DNA/RNA co-threading configuration (I/I_0=16.2%) was between those of dC35 (I/I_0=18.6%) and dC14-rC5 (I/I_0=13.2%). This result indicates that the blocking level varies based on the number of RNA nucleotides occupying the pore stem. **c.** Cartoons showing the RNA positions (top) and blocking level histogram (bottom) showing the discrimination of the streptavidin-attached chimeras dC5-rC5, dC8-rC5, dC11-rC5, and dC14-rC5 in the nanopore. Unlike the chimeras in b, these chimeras possess a common 5-nt poly(rC) RNA that partially occupies the pore stem but at different locations in the pore. Upon trapping, the RNA of dC5-rC5 is located across the constrictive site around the inner opening of the β-barrel (in the middle of the pore). This configuration resulted in the lowest blocking level (I/I_0=14.3%) among the four chimeras. This condition is in contrast to the same RNA in dC14-rC5, which was located near the *trans* entrance and generated the highest blocking level (I/I_0=16.2%). By comparison, the same RNAs in dC8-rC5 and dC11-rC5 were shifted to the middle of the pore stem. The resulting blocking levels for the two RNA locations (I/I_0=15.6% for dC8-rC5 and I/I_0=15.2% for dC11-

rC5) were between those of dC5-rC5 (I/I_0=14.3%) and dC14-rC5 (I/I_0=16.2%). This result indicates that the location of RNA trapped in the pore stem can further regulate the blocking level of the chimeras. Overall, the findings suggest that the configurations of full and partial occupations of the nanopore by an RNA fragment in a DNA strand, as well as its location in the pore, can be discriminated by the nanopore conductance variation.

References

1. Stoddart D., Heron A. J., Mikhailova E., Maglia G., Bayley H. Single-nucleotide discrimination in immobilized DNA oligonucleotides with a biological nanopore. *Proc. Natl. Acad. Sci. USA* **106**, 7702-7707 (2009).
